# Clinical Profile and Outcome in Patients with Coronary Slow Flow Phenomenon

**DOI:** 10.1155/2019/9168153

**Published:** 2019-05-07

**Authors:** Xiaogang Zhu, Hua Shen, Fei Gao, Sijing Wu, Qian Ma, Shuo Jia, Ziwei Zhao, Shan Tong, Zhihao Zhang, Yujie Zhou

**Affiliations:** ^1^Department of Cardiology, Beijing Anzhen Hospital, Capital Medical University, Beijing Institute of Heart Lung and Blood Vessel Disease, Beijing 100029, China; ^2^Department of Cardiology, Fuxing Hospital, Capital Medical University, Beijing 100038, China; ^3^JetMed (Beijing) Co., Ltd., Beijing 100024, China

## Abstract

The coronary slow flow phenomenon (CSFP) is a poorly recognized clinical entity characterized by delayed distal vessel opacification in the absence of epicardial coronary stenosis and presently lack of specific data on the clinical profile and outcome. We investigated a cohort of 429 patients who fulfilled the criteria for CSFP to explore the clinical feature, outcome, and risk factor of prognosis. Two teams (clinical center and core lab) were blind to patient data for the assessment of coronary angiograph using corrected thrombolysis in myocardial infarction (TIMI) frame count (CTFC). The study cohort consisted of 429 patients (294 men, 68.5%), aged from 30 to 78 years (mean, 54 years). Two hundred patients (46.6%) out of 429 patients had a history of hypertension, 72 (16.8%) had diabetes mellitus, and 222 (51.7%) had dyslipidemia. All the rates of agreement between two teams in evaluating whether normal flow (CTFC ≤ 27 frames) or slow flow (CTFC > 27 frames) were moderate (0.40 < *κ* < 0.75) for the three arteries. Follow-up (mean, 3.8 years) was done for 421 patients (98.1%). The major adverse cardiovascular events (MACE) occurred in 39 patients (9.3%) out of 421 patients. Multivariate analysis showed that the risk of MACE approximately doubles with age >50 years (hazard ratio (HR) = 2.2, 95% CI: 1.0 to 4.9, and *P*=0.042), hypertension (HR = 2.1, 95% CI: 1.1 to 4.2, and *P*=0.021), and dyslipidemia (HR = 2.0, 95% CI: 1.0 to 3.9, and *P*=0.042). CSFP affects predominantly patients at middle age and above but can occur in any age group; CSFP should be more concerned, particularly in patients >50 years old with hypertension and dyslipidemia.

## 1. Introduction

The coronary slow flow phenomenon (CSFP) is characterized by the slow antegrade passage of dye through one or more vessels of the coronary tree without stenosis during coronary angiography. Since it was firstly described by Tambe et al. in 1972 [1], many studies focused on the risk factors of CSFP have been reported; however, there is a paucity of specific data on the clinical profile and outcome of these patients. Clinical center and angiographic core laboratory were blind to patient data for the assessment of coronary angiograph using corrected thrombolysis in myocardial infarction (TIMI) frame count (CTFC) in this study. Moreover, the clinical profile and outcome of the patients were evaluated, and the prognostic factor was explored using proportional hazards. In the present study, it was sought to investigate the clinical feature and prognosis of the patients with CSFP.

## 2. Materials and Methods

### 2.1. Study Population

In this study, 1,67,494 consecutive patients who underwent coronary angiography in our clinical center between 2009 and 2017 were assessed by TIMI flow grade and excluded if they had any known or documented ischemic heart disease (previous or current infarction, revascularization, and ≥20% diameter coronary stenosis), coronary ectasia, coronary artery spasm, coronary myocardial bridge, valvular heart disease (more than mild), cardiomyopathy, heart failure, and malignancy, as well as unavailable angiographic or clinical data. Then, 484 patients with TIMI grade 2 flow (requiring three or more beats to opacify the distal vessel) in at least one major vessel were preliminarily subsumed.

This study was approved by our local research ethics committee and conducted in accordance with the ethical principles of the Declaration of Helsinki. Informed consent was obtained from all the participants.

### 2.2. Assessment of Coronary Angiogram (CTFC)

**Figure 1:**
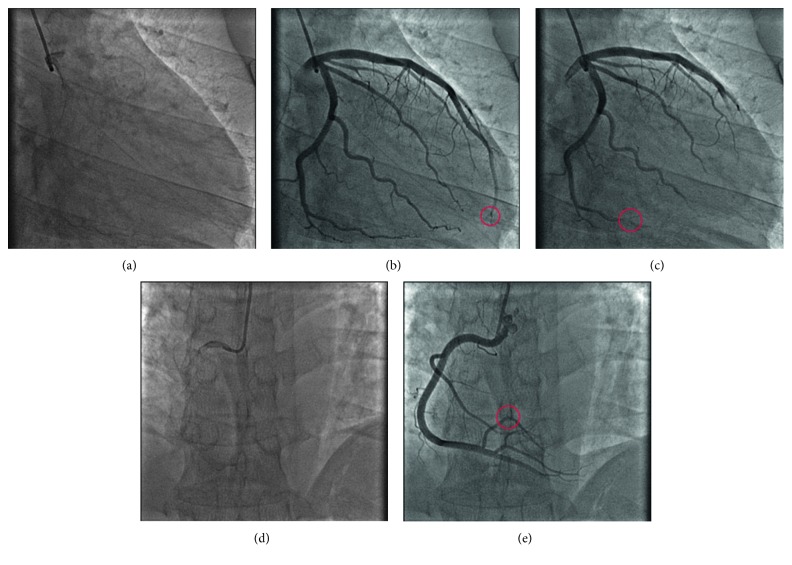
TIMI frame count and the definition of the first frame and the last frame. (a) The first frame of the LAD and LCX in which dye first touches both borders at the origin of the two arteries; (b) the last frame of the LAD in which dye first enters the distal landmark branch: the distal-most bifurcation of the LAD (red circle), usually at the apex of the heart, like the “pitchfork” in this case; (c) the last frame of the LCX in which dye first enters the distal bifurcation of the segment with the longest total distance (red circle); (d) the first frame of the RCA in which dye first touches both borders at the origin of the RCA; (e) the last frame of the RCA in which dye first enters the distal landmark branch: the distal landmark is the first branch arising from the posterior lateral extension of the RCA after the origin of the posterior descending artery (red circle). LAD = left anterior descending coronary artery; LCX = left circumflex artery; RCA = right coronary artery.

These patients preliminarily subsumed were reassessed by clinical center and angiographic core laboratory, blind method, using CTFC described by Gibson et al. [2]. For TIMI frame counting, the first frame was defined as the frame in which dye first completely filled the entrance of the artery with antegrade flow, and the last frame was defined as the frame in which dye first entered the distal landmark branch (Figure 1). The left anterior descending coronary artery (LAD) frame counts were divided by 1.7 for correction of the longer length, and all the films should be corrected at 30 frames per second (fps). The CTFC above 27 frames for at least one among three major vessels was defined to CSFP, only if the qualitative results from clinical center and angiographic core laboratory were consistent. Eventually, a cohort of 429 patients fulfilled the criteria for CSFP.

### 2.3. Clinical Data Collection

Demographic data regarding age, sex, body mass index (BMI), cardiovascular risk factors (hypertension, diabetes, dyslipidemia, cigarette smoking, etc.), and clinical presentation were recorded. Data of electrocardiograph (ECG) and echocardiography were collected. Then, the left ventricular mass index (LVMI) was calculated according to Devereux's formula [3], and left ventricular hypertrophy (LVH) was defined as LVMI >95 g/m^2^ in females or LVMI >115 g/m^2^ in males [4].

### 2.4. Follow-Up and Outcome

Follow-up evaluation was attempted for all patients by telephone or visit, which included major adverse cardiovascular events (MACE), concomitant symptoms, and long-term medications after discharge. In this study, MACE was defined as cardiac death, nonfatal myocardial infarction (MI), revascularization, hospitalization due to unstable angina pectoris, and nonfatal stroke. In case the patient had died, an attempt was made to identify the cause (cardiac and noncardiac).

### 2.5. Statistical Analysis

Data were expressed as mean and standard deviation (SD) or frequency percents. Analyses were conducted on the raw data. Variable differences were assessed by paired *t*-test for continuous variables and *χ*^2^-test for discrete variables; crosstab analysis and kappa value (*κ* statistic) were used in the consistency evaluation. The survival follow-up data were analyzed by univariate and multivariate Cox proportional hazards regression. Missing data were omitted, where samples with invalid data are discarded from further analysis. All analyses were conducted with SAS version 9.3 software (SAS Institute Inc., Cary, NC). Two-tailed *P* values less than 0.05 were considered to be statistically significant.

## 3. Results

### 3.1. Clinical Feature

**Figure 2:**
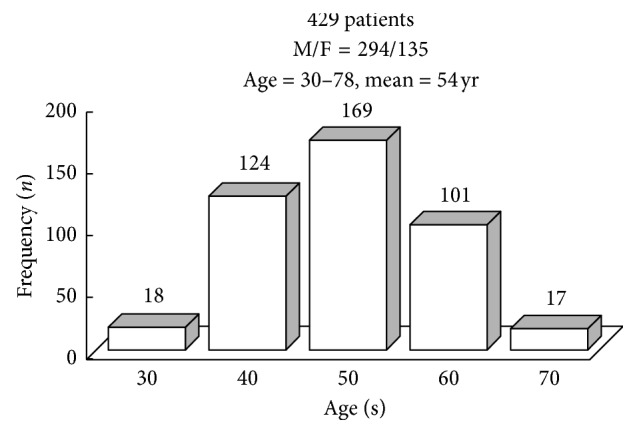
Age distribution of study cohort. Sixty-seven percent of patients were >50 years old. F = female; M = male.

The study cohort consisted of 429 patients (294 men, 68.5%). In the initial evaluation, the age range was from 30 to 78 years (mean, 54 years). As demonstrated in Figure 2, 67% of patients were >50 years old. Baseline characteristics are displayed in Table 1. The mean body mass index (BMI) was 26.3 kg per square meter. Two hundred patients (46.6%) of 429 had a history of hypertension, 72 (16.8%) had diabetes mellitus, 222 (51.7%) had dyslipidemia, 205 (47.8%) were previous or current smokers, 102 (23.8%) were moderate to heavy alcohol drinkers, 12 (2.8%) had obstructive sleep apnea-hypopnea syndrome, 68 (15.9%) had family history of coronary artery disease, and 421 (98.1%) had symptom of chest pain; only 22 (5.1%) were diagnosed with “acute coronary syndrome” at the time of discharge.

### 3.2. Electrocardiography and Echocardiography

The 12-lead ECG at the time of the initial admission was reviewed in 403 patients and demonstrated complete right bundle branch block in 10 (2.5%) out of 403 patients, complete left bundle branch block in 2 patients (0.5%), ST-segment depression in 36 patients (8.9%), and ST-segment elevation in 5 patients (1.2%). Nonspecific ST-T-wave abnormalities were noted in 74 patients (18.4%), and 276 patients had a normal ECG (69%).

The echocardiographic findings in our study cohort were summarized in 338 patients. Average left ventricular end-diastolic diameter was 47.1 mm (range, 35 to 55 mm). Left ventricular ejection fraction was assessed quantitatively, and the ejection fraction averaged 66% (range, 53% to 80%). The echocardiography demonstrated regional wall motion abnormality in 13 (3.8%) out of 338 patients, and 28 patients (8.3%) were assessed as left ventricular hypertrophy.

### 3.3. Coronary Angiography

#### 3.3.1. TIMI Flow Grades for Coronary Arteries by Clinical Center and Core Lab

As to the left anterior descending coronary artery (LAD), 215 patients (50.1%) out of 429 were assessed as TIMI grade 2 flow by clinical center and 287 (66.9%) by angiographic core laboratory (*P* < 0.001). The patients with TIMI grade 2 flow for the left circumflex artery (LCX) amounted to 327 (76.2%) assessed by clinical center and 92 (21.4%) by angiographic core laboratory (*P* < 0.001). As for the right coronary artery (RCA), 317 patients (73.9%) out of 429 patients were assessed as TIMI grade 2 flow by clinical center and 157 (36.6%) by angiographic core laboratory (*P* < 0.001). The results of TIMI flow grades for coronary arteries are displayed in Table 2.

#### 3.3.2. Agreement in Assessment of Conventional TIMI Flow Grades between Clinical Center and Core Lab

As mentioned above, TIMI flow grades were evaluated by two teams (clinical center and core lab), respectively. Furthermore, the rate of agreement between clinical center and core lab in the evaluation of TIMI flow (TIMI grade 2 flow or TIMI grade 3 flow) was assessed with use of *κ* statistic (range of values, −1 to +1). The value of *κ* > 0.75 indicates excellent agreement between two observers; however, value <0.40 indicates poor agreement. Agreement was poor in assessment of TIMI flow for LAD, with a 62.3% rate of agreement between clinical center and core lab (*κ* = 0.24 ± 0.05). There was a poor rate (42.0%) of agreement in assessment of TIMI flow for LCX (*κ* = 0.11 ± 0.05). Furthermore, for assessment of TIMI flow for RCA, the rate of agreement was also poor at 54.8% (*κ* = 0.20 ± 0.05).

#### 3.3.3. CTFC for Coronary Arteries by Clinical Center and Core Lab

The CTFC for LAD averaged 37 (SD, 12) frames by clinical center and 44 (SD, 14) frames by core lab (*P* < 0.001). The mean CTFC for LCX was 47 (SD, 17) frames by clinical center and 54 (SD, 21) frames by core lab (*P* < 0.001). The CTFC for RCA averaged 47 (SD, 22) frames by clinical center, which was significantly higher than that (averaged 42 (SD, 21) frames) assessed by core lab (*P* < 0.001). The results of CTFC for coronary arteries are displayed in Table 3.

#### 3.3.4. Agreement in Evaluating Whether It Is Normal Flow or Slow Flow (Using CTFC) between Clinical Center and Core Lab

The coronary flow result whether normal flow (CTFC ≤ 27frames) or slow flow (CSFP, CTFC > 27frames) was evaluated by clinical center and core lab, and the rate of agreement between clinical center and core lab in the evaluation of CSFP or not was also assessed with use of *κ* statistic. Agreement was moderate in assessment of CSFP or not for LAD, with a 92.3% rate of agreement between clinical centers and angiographic core laboratory (*κ* = 0.65 ± 0.05). There was a moderate rate (92.8%) of agreement in assessment of CSFP or not for LCX (*κ* = 0.48 ± 0.05). Furthermore, for assessment of CSFP or not for RCA, the rate of agreement was also moderate at 87.9% (*κ* = 0.62 ± 0.05). According to the identical results assessed by clinical center and core lab, 429 patients had slow flow coronary phenomenon in at least one major vessel.

### 3.4. Follow-Up

Follow-up was done for 421 patients (98.1%) out of 429 patients, with a mean duration of 3.8 years (range, 0.7 to 9.3 years). Sixteen patients (3.8%) returned to our center; for the remaining 405 patients, follow-up information was obtained by phone with the patient in the flesh if alive and with the next of kin if passed away. Five patients (1.2%) had died. Death was attributed to cardiac cause in 3 patients (sudden cardiac death) and noncardiac cause in 2 patients (lung cancer; melanoma). Among the 416 survivors, 154 patients had relapses of angina pectoris, 28 had hospitalization due to unstable angina pectoris, 11 had repeated coronary examinations (among these patients, 4 still showed CSFP), 12 had recurrent syncope, 1 had nonsustained ventricular tachycardia, 9 had atrial fibrillation, 3 patients had nonfatal myocardial infarction (MI), none had revascularization, and 11 had stroke (10 was ischemic; 1 was hemorrhagic and ischemic). For some patients, there were some overlaps or concurrences for these events.

**Figure 3:**
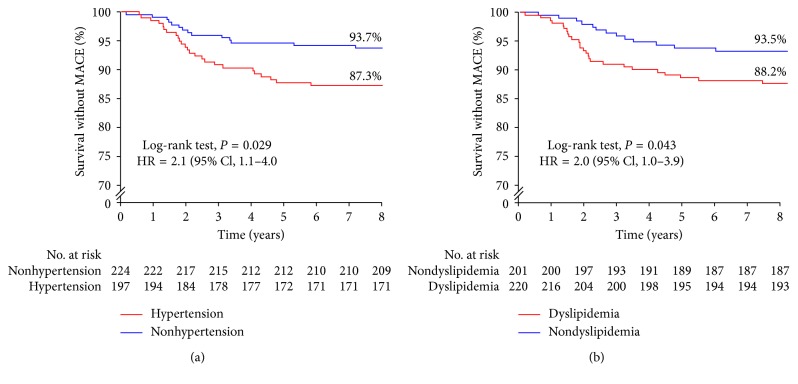
Survival (without MACE) curves in relation to hypertension (a) and dyslipidemia (b). MACE = major adverse cardiovascular events.

Overall, MACE with a mean duration of 2.5 years (range, 0.2 to 7.2 years) after discharge occurred in 39 patients (9.3%) out of 421 patients, which was a composite of cardiac death, nonfatal MI, revascularization, hospitalization due to unstable angina pectoris, and nonfatal stroke in the study. Univariate analysis demonstrated that MACE with CSFP was not significantly related to sex, age, body mass index, diabetes mellitus, smoking, drinking, or medications. However, MACE with CSFP was related to hypertension (hazard ratio (HR) = 2.1, 95% CI: 1.1 to 4.0, and *P*=0.029) and dyslipidemia (HR = 2.0, 95% CI: 1.0 to 3.9, and *P*=0.043) (Figure 3). Multivariate analysis showed that the risk of MACE was significantly independently associated with age >50 years (HR = 2.2, 95% CI: 1.0 to 4.9, and *P*=0.042), hypertension (HR = 2.1, 95% CI: 1.1 to 4.2, and *P*=0.021), and dyslipidemia (HR = 2.0, 95% CI: 1.0 to 3.9, and *P*=0.042).

## 4. Discussion

Although dozens of formal definitions have been put forward, the CSFP essentially consists of the delayed progression of contrast injected into an epicardial coronary artery without stenosis during coronary angiography. In virtue of discrepancies in defining CSFP, incidence range of 1–7% in serial angiographies, and up to 5% in cases of acute coronary syndromes, has been reported [1, 5]. Most previous patient series were hampered by heterogeneity of included patients, angiographic inclusion criteria; however, our study emphasizes that the CSFP, an independent clinical entity, is “primary” CSFP, which should be distinguished from coronary reperfusion therapy-induced slow flow, or other “secondary” causes of coronary slow flow. These causes include coronary artery ectasia, coronary artery spasm, pulmonary arterial hypertension, valvular heart disease, cardiomyopathy, connective tissue disorders, or heart failure. Slightly unlike Beltrame criteria for diagnosing primary coronary slow flow [6], we strictly exclude obstructive epicardial coronary artery disease (no lesions ≥20%). So, this study aims to evaluate clinical profile and prognosis of the patients with primary CSFP.

CSFP is more common in middle-aged and old men (M : F, 2.2 : 1) in our study; some studies have regarded male gender as a predictor of CSFP, while others have found no relation between sex and CSFP [7]. CSFP is most common in patients admitted with symptom of chest pain; rest or mixed-pattern angina with durations of symptom from several minutes to tens of minutes is a distinguishing characteristic of CSFP. Most importantly, CSFP has been delineated to be associated with life-threatening arrhythmias and sudden cardiac death [8, 9]. Generally speaking, in all but severe cases of unstable hemodynamics, no special physical findings are in patients with CSFP.

To assess coronary flow, as we all know, both TIMI flow grade and CTFC can be used. TIMI flow grade is a valuable and widely used qualitative measure in angiographic clinical and trials, while it is limited by its variable, categorical, and subjective nature [2]. On the contrary, the TIMI frame-counting method (CTFC) is reproducible, quantitative, and relatively objective. In our study, two teams (clinical center and core lab) were blind to patient data for the assessment of coronary angiograph using corrected TIMI frame count (CTFC). It was demonstrated that the results assessed by CTFC method have higher frequency of agreement than that by TIMI flow grade method between clinical center and angiographic core laboratory, which proved CTFC method to be more reproducible and reliable. So, different from Beltrame criteria [6] for diagnosing primary coronary slow flow, which defined CSFP by either TIMI 2 flow or CTFC > 27frames, we define CSFP only by CTFC method (CTFC > 27frmes).

In addition, some studies showed that Doppler echocardiographic-derived coronary flow velocity had prognostic value [10, 11]; however, due to anatomic factors and technological limitation (e.g., the application was confined to LAD), the noninvasive demonstration of coronary flow pattern (transthoracic Doppler echocardiography, TTDE) does not widely apply to the assessment of coronary flow, including the CSFP.

Little is known about the prognosis of true primary CSFP, because the most published literature has included patients with known heart failure, near-normal coronary arteries (<40% stenosis), and other unexplained diseases [12, 13], and as a result, their outcome was not substantially the same as that observed in this study. In this study, we have tried to exclude patients with a possible secondary form of CSFP and other disease states as has been said before by sticking to very strict inclusion and exclusion criteria. Furthermore, in our study, two teams (clinical center and core lab) participate in the assessment of patient imaging, which can guarantee the accuracy of outcome in the maximum extent.

As previously mentioned, there is a paucity of specific data on the outcome of the patients with CSFP, not only that, there is considerable controversy regarding the prognosis. Sadamatsu et al. and Chaudhry et al. reported that patients with CSFP had a favorable long-term prognosis [13, 14], while Fragasso et al. investigated 12 patients with CSFP by an averaged follow-up of 15 years and thought that patients with CSFP were associated with a worse cardiac prognosis and should be carefully followed-up [15]. Besides that, the number of patients reported was limited (from a dozen to over a hundred); so, this phenomenon (CSFP) remains poorly understood. Our study, compared with those published reports, had relatively adequate sample size and explored the risk factor of prognosis for the first time in forever. The observed overall major adverse cardiovascular events (MACE) occurred in 39 (9.3%) out of 421 patients. The risk of MACE by multivariate analysis was significantly independently associated with age >50 years (HR = 2.2, 95% CI: 1.0 to 4.9, and *P*=0.042), hypertension (HR = 2.1, 95% CI: 1.1 to 4.2, and *P*=0.021), and dyslipidemia (HR = 2.0, 95% CI: 1.0 to 3.9, and *P*=0.042). These findings suggest that CSFP might have a prolonged process in the early phase of the disease, when patients are relatively younger. However, patients at middle age and above, with hypertension and dyslipidemia, have the worse prognosis.

CSFP may be a combination of multifactorial abnormality in which inflammatory status, endothelial dysfunction, subclinical atherosclerosis, as well as structural and functional abnormalities in the coronary microcirculation play an important role, resulting in transient or persistent myocardial hypoperfusion. To date, treatment is not well defined and is mainly directed at influencing functional obstruction in arterioles (<200 *μ*m) with dipyridamole or mibefradil [16, 17], controlling abnormal cholesterol and vascular inflammation with statins [18, 19], and improving endothelial function as well as alleviating symptoms [20, 21]. In addition, hypertension and dyslipidemia need better control in patients with CSFP, on the basis of our finding.

Our study has several limitations. This is a single cohort study (lack of control group) that required data collection over 9 years, during which few patients returned for follow-up, and a large proportion was contacted by telephone. As a result of the retrospective design of this study, the hypothesis that CSFP may result in myocardial hypoperfusion was not tested, which could have been achieved by cardiac magnetic resonance imaging or radionuclide myocardial perfusion. Although we have attempted, to the best of our ability, to reduce the deficiency, these limitations are innate to the retrospective design of the study. Future work is encouraged to initiate further large-scale prospective studies that reveal the pathogenesis involved in CSFP, better characterize this phenomenon, and most importantly, investigate therapeutic approaches and long-term prognosis.

To sum up, the coronary slow flow phenomenon (CSFP) characterized by delayed distal vessel opacification without epicardial coronary stenosis, as assessed quantitatively using the corrected TIMI frame count (CTFC), should be more concerned. This study describes the clinical, electrocardiographic, and echocardiographic features as well as coronary angiographic presentation of patients with CSFP. Although these features of CSFP are nonspecific, our study reveals that the presence of age above 50 years, hypertension, and dyslipidemia, is associated with adverse outcome. The pathogenesis, therapeutic approach, and long-term prognosis involved in CSFP are not fully interpreted and are the directions of much ongoing research.

## Figures and Tables

**Table 1:** Baseline characteristics of the patients with CSFP.

Feature	Overall (*n*=429) Mean (SD)/*n* (%)
Age (yrs)	54.4 (8.9)
Male	294 (68.5)
BMI (kg/m^2^)	26.3 (3.5)
Hypertension	200 (46.6)
Diabetes mellitus	72 (16.8)
Dyslipidemia	222 (51.7)
Current or previous smoker	205 (47.8)
Moderate to heavy alcohol drinker	102 (23.8)
Obstructive sleep apnea-hypopnea syndrome	12 (2.8)
Family history of coronary artery disease	68 (15.9)
Symptom of chest pain	421 (98.1)
Diagnosis of acute coronary syndrome	22 (5.1)

CSFP = coronary slow flow phenomenon; BMI = body mass index.

**Table 2:** TIMI flow grades for coronary arteries by clinical center and core lab.

Coronary artery	Clinical center *n* (%)	Core lab *n* (%)	*P* value
LAD, TIMI grade 2	215 (50.1)	287 (66.9)	<0.001
LCX, TIMI grade 2	327 (76.2)	92 (21.4)	<0.001
RCA, TIMI grade 2	317 (73.9)	157 (36.6)	<0.001

TIMI = thrombolysis in myocardial infarction; LAD = left anterior descending coronary artery; LCX = left circumflex artery; RCA = right coronary artery.

**Table 3:** CTFC for coronary arteries by clinical center and core lab.

Coronary artery	Clinical center Mean (SD)	Angiographic core laboratory Mean (SD)	*P* value
LAD, frames	37 (12)	44 (14)	<0.001
LCX, frames	47 (17)	54 (21)	<0.001
RCA, frames	47 (22)	42 (21)	<0.001

CTFC = corrected thrombolysis in myocardial infarction (TIMI) frame count; LAD = left anterior descending coronary artery; LCX = left circumflex artery; RCA = right coronary artery.

## Data Availability

The data used to support the findings of this study are available from the corresponding author upon request.
